# A protocol to count *Cryptosporidium* oocysts by flow cytometry without antibody staining

**DOI:** 10.1371/journal.pntd.0007259

**Published:** 2019-03-20

**Authors:** Karine Sonzogni-Desautels, Thomas Z. Di Lenardo, Axel E. Renteria, Marc-André Gascon, Timothy G. Geary, Momar Ndao

**Affiliations:** 1 National Reference Centre for Parasitology, Research Institute of the McGill University Health Centre, Montreal, QC, Canada; 2 Program in Infectious Diseases and Immunity in Global Health, Research Institute of the McGill University Health Centre, Montreal, QC, Canada; 3 Institute of Parasitology, McGill University, Ste Anne de Bellevue, QC, Canada; 4 Department of Medicine, Division of Experimental Medicine, McGill University, Montreal, QC, Canada; 5 Department of Medicine, Division of Infectious Diseases, McGill University, Montreal, QC, Canada; PUCRS, BRAZIL

## Abstract

Cryptosporidiosis caused by the protozoan parasites *Cryptosporidium hominis* and *C*. *parvum*, threatens the lives of young children in developing countries. In veterinary medicine, *C*. *parvum* causes life-threatening diarrhea and dehydration in newborn dairy calves. Protocols to detect *Cryptosporidium* spp. oocysts using flow cytometry have been reported; however, these protocols use antibodies against the parasite and typically focus on detection of oocysts, not quantification. These techniques are not well-suited for studies that generate large variations in oocyst burdens because the amount of antibody required is proportional to the number of oocysts expected in samples. Also, oocysts are lost in washes in the staining protocol, reducing accuracy of oocyst counts. Moreover, these protocols require costly fluorochrome-conjugated monoclonal antibodies and are not optimal for studies involving large numbers of samples. Here we present an optimized protocol for purifying oocysts from mouse stool and intestine samples combined with a reliable method to quantify oocysts in a relatively pure population without the need for antibody staining. We used morphology (SSC-A vs FSC-A) and the innate characteristics of *C*. *parvum* oocysts compared to fecal and intestinal contaminants to develop a two-step gating strategy that can differentiate oocysts from debris. This method is a fast, reliable, and high-throughput technique to promote research projects on *C*. *parvum* infections in mice and potentially other animal hosts.

## Author summary

Diarrheal diseases are the second leading cause of death in children < 5 years old. Cryptosporidiosis caused by the unicellular parasite *Cryptosporidium* spp. is one of these diarrheal diseases. *C*. *hominis* and *C*. *parvum* cause moderate-to-severe diarrhea and dehydration that threaten the lives of young children in developing countries. Flow cytometry is a state-of-the-art technique to detect *Cryptosporidium* spp. oocysts, the infectious form of the parasite. Reported protocols typically focus on detection of oocysts using antibody staining. However, these techniques present several complications: oocysts are lost in washes used in the staining protocol and the amount of antibody required is proportional to the number of oocysts expected in samples; thus, parasite burden needs first to be estimated by optical microscopy. In addition, these protocols require costly antibodies. We developed a reliable method to quantify *Cryptosporidium* spp. oocysts in a relatively pure population without the need for antibody staining. We used known characteristics of the structure of oocysts to develop a strategy that can differentiate oocysts from debris. This method is fast, affordable and reliable and will facilitate pre-clinical projects on interventions to treat or prevent *Cryptosporidium spp*. infection.

## Introduction

Cryptosporidiosis is an ubiquitous disease particularly common in young children in developing countries [1]. A prospective case-controlled study conducted in sub-Saharan Africa and South Asia on children with moderate-to-severe diarrhea showed that *Cryptosporidium spp*. was the second most prevalent pathogen among infants 0–11 months old [2]. Infection with this parasite was also associated with an increased risk of death in children 12–23 months old [2]. Immunocompetent people are also at risk and many outbreaks have been reported in industrialized countries following oocyst contamination of drinking or recreational water [3]. From a veterinary perspective, this disease affects most pre-weaned dairy calves and causes significant economic losses [4–6]. Mortality rates of 10–16% have been reported in *C*. *parvum*-infected newborn dairy calves co-infected with other enteric pathogens [7, 8].

Humans are usually infected with *C*. *hominis*, the human-specific species, while both humans and calves can be infected with the zoonotic species *C*. *parvum* [3]. Cattle and calves can also be infected with *C*. *bovis* and *C*. *andersoni*; nevertheless, newborn dairy calves are predominantly infected with *C*. *parvum* [4, 9, 10]. Oocysts of *C*. *hominis* and *C*. *parvum* are similar in morphology [3, 9, 11, 12]. Efficient *C*. *parvum* infection models have been established in mice [13–16], but not for *C*. *hominis* [9, 12]. As a result, *C*. *parvum* infection models in mice are commonly used to study human and bovine cryptosporidiosis. A murine model of *C*. *parvum* infection is used in our laboratory for drug and vaccine discovery [13–15], in which the ability to quantify oocysts purified from stool or intestine of infected mice is essential to determine if a drug or vaccine decreases parasite burden [15].

Protocols to detect *Cryptosporidium spp*. oocysts by flow cytometry using antibody staining are available [17–20]. However, these protocols have limitations for oocyst quantification. First, the oocyst burden of infected control mice compared to mice receiving a therapeutic treatment can vary by more than five orders of magnitude [15]. This can result in under-stained high burden samples or a waste of antibody by over-staining low burden samples. Second, oocysts may be lost in the washing steps of the antibody staining protocols. For studies in which intra-group and inter-group variations are crucial for data analysis, this bias can lead to incorrect data interpretations. Third, when staining large numbers of samples generated in an experimental study, the cost can become problematic. For these reasons, we optimized the protocol to purify oocysts from mouse stool and intestine samples and developed a novel selective gating strategy for quantifying *C*. *parvum* oocysts in relatively pure samples by flow cytometry without using antibodies.

## Materials and methods

### Mouse infection and oocyst purification

*C*. *parvum* (field strain) oocysts collected from infected calves were generously provided by Prof. Dwight D. Bowman (Cornell University, Ithaca, NY, USA) and propagated in C57BL/6 IFNγR-KO mice as described [14–15]. Briefly, oocysts kept at 4°C in potassium dichromate (K_2_Cr_2_O_7_, Sigma-Aldrich, Oakville, ON, Canada) were washed three times with phosphate-buffered saline (PBS) and 3,000 oocysts in 100 μL PBS were used to infect 6-8-week-old mice by oral gavage.

Oocysts were purified from intestines of infected mice as described [15]. Briefly, mice were sacrificed 10 days post-infection and the entire intestine from duodenum to rectum was ground in a 50 mL sample container with 10 mL 0.04% v/v Tween 20 in PBS. Intestine samples were then incubated with 0.05 g sputasol (dry mixture of 10% DTT, 76% NaCl, 2% KCl, 10% Na_2_HPO_4_ and 2% KHPO_4_) for 90 min at 4°C on rotary mixers and transferred to 50 mL Falcon tubes (BD-Canada, Mississauga, ON, Canada) for centrifugation at 2,500 x g for 10 min at 4°C. The supernatant was then removed and the pellet resuspended in 8 mL 0.04% (v/v) Tween20 in distilled water and 4 mL diethyl ether (Sigma-Aldrich) and centrifuged again. The supernatant and fatty layer were removed and the pellet was washed with 20 mL cold distilled water and centrifuged again. The supernatant was discarded and the pellet resuspended in 20 mL saturated NaCl. After thorough vortexing, samples were overlayed with 5 mL cold distilled water. The samples were then centrifuged, and 4 mL of the interphase was collected, diluted with an equal volume of distilled water and aliquoted into Eppendorf tubes. After thorough mixing, tubes were centrifuged at 16,400 x g for 30 min at 4°C. Then, 90% of the supernatant was discarded and the pellets were resuspended in the remaining solution. Samples from the same mouse were pooled and the volume adjusted to 1 mL with distilled water. A similar protocol was used to purify oocysts from stool of infected mice [15]. Oocysts were kept at 4°C in ddH_2_O for short-term storage (few weeks) or in 2.5% w/v K_2_Cr_2_O_7_ for long-term storage (maximum 4 months).

### Ethics statement

The animal use protocol (#2015–7664) was approved by the Glen Facility Animal Care Committee of the Research Institute of the McGill University Health Centre. All experiments were conducted in accordance with the guidelines of the Canadian Council on Animal Care.

### Monoclonal antibody staining

The procedure for monoclonal antibody staining of oocysts was adapted from Barbosa et al. [17]. Briefly, oocysts purified from intestines of infected C57BL/6 IFNγR-KO mice and kept in 2.5% w/v K_2_Cr_2_O_7_ were washed three times in PBS and centrifuged at 10,000 x g for 5 min at 4°C. Oocysts were counted using a hemocytometer and diluted in PBS to create several stock oocyst solutions from 10^3^ to 5X10^6^ oocysts/mL. Samples > 10^4^ oocysts/mL were recounted using a hemocytometer to confirm concentration; the 10^3^ oocysts/mL sample was not recounted due to the limit of detection of the hemocytometer and its concentration was estimated from 1:10 dilution of the 10^4^ oocysts/mL sample. Then, 100 μL of each oocyst solution was incubated for 30 min at room temperature with 1 μL mouse anti-*Cryptosporidium* Alexa Fluor 488 monoclonal antibody clone BEL 0126 (AbD Serotec, Raleigh, NC, USA) at a final antibody concentration of 0.5 μg/mL. As a washing step, 900 μL 1% w/v bovine serum albumin (BSA) in PBS was added to each sample prior to centrifugation at 10,000 x g for 5 min at room temperature; the supernatant was discarded and oocysts were resuspended in 20 μL 1% BSA in PBS. For each oocyst concentration, samples were done in triplicate in each of three independent experiments.

### Sample preparation for flow cytometry

To quantify *C*. *parvum* oocysts, 20 μL of a solution of purified oocysts from mouse intestines (1:10 dilution considering that total volume = 200 μL) or 4 μL of a solution of purified oocysts from mouse stool (1:50 dilution considering that total volume = 200 μL) were diluted with 60 μL or 76 μL PBS, respectively. Samples were incubated overnight with 100 μL 8% paraformaldehyde (Sigma-Aldrich). Paraformaldehyde only fully inactivates oocysts at room temperature, but some excystation may occur. We recommend incubation at 4°C and following standard biosafety measures (i.e., wear gloves and standard disinfection of flow cytometer with bleach after acquisition). Then, 20 μL CountBright Absolute Counting Beads (0.54 x 10^5^ beads/50 μL; Thermo Fisher Scientific, Burlington, ON, Canada) were added to each sample prior to analysis by flow cytometry (total volume = 200 μL).

### Flow cytometry analysis

To prove that this method for counting oocysts without staining is independent of the model of flow cytometer, we ran the same samples on 3 flow cytometers to compare the efficiency of the gating strategy. Samples were analyzed using a BD LSRFortessa, a BD LSRFortessa X-20, and a BD FACSCanto II flow cytometer using BD FACSDiva software (BD Biosciences, San Jose, CA, USA). The forward scatter area (FSC-A) and side scatter area (SSC-A) represent relative measures of size and complexity, respectively. For each flow cytometer, the Alexa Fluor 488 channel [bandpass filter (BP): 530/30] of the 488 nm laser was used. For the BD LSRFortessa, the PerCP channel [BP: 710/50] of the 488 nm laser was used. For the BD LSRFortessa X-20 flow cytometer, the PerCP channel [BP: 695/40] of the 488 nm laser was used; and for the BD FACSCanto II, the PerCP channel [BP: 670–735] of the 488 nm laser was used.

### Data analysis

Flow cytometry data were analysed using FlowJo v10 (FlowJo, LLC, Ashland, OR, USA). Bar graph and scattered dot plot were generated using GraphPad Prism Version 7 (GraphPad Software, La Jolla, CA, USA). Linear regression and One-way ANOVA with Tukey’s multiple comparisons post-test were used. Differences with p < 0.05 were considered statistically significant; * = p < 0.05, ** = p < 0.01, *** = p < 0.001 and **** = p < 0.0001.

## Results

### Identification of *C*. *parvum* oocysts using monoclonal antibody staining

**Fig 1:**
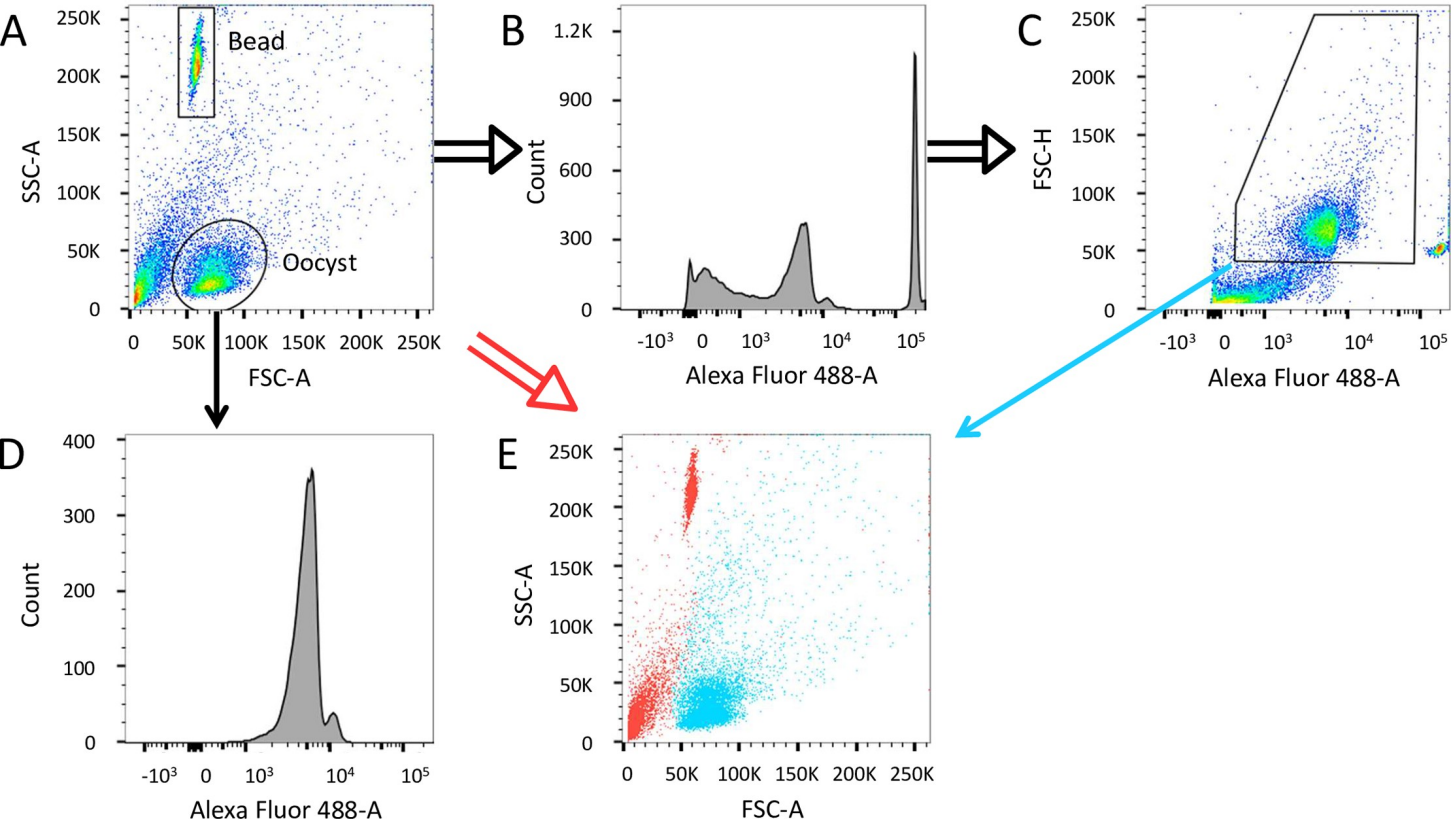
Staining of oocysts with a mouse anti-*Cryptosporidium* Alexa Fluor 488 monoclonal antibody. (A to E) Representation of an Alexa 488 stained sample containing 10^4^ oocysts. (A) A representative plot of oocysts purified from mouse intestine. The oval Oocyst gate encompasses the expected location of the *C*. *parvum* oocysts on the plot of SSC-A versus FSC-A based on a positive control of purified oocysts. Histograms showing the distribution of events positive for Alexa Fluor 488 emission on the ungated sample (B) and the Oocyst gate (D). (C) A pseudo-coloured dot plot of FSC-H versus Alexa 488 with the recommended gate for Alexa 488+ oocysts. (E) Overlay of Alexa 488+ oocysts from panel C (blue) and negative events (red) of the ungated sample.

Fig 1 shows representative flow cytometry plots for a sample containing 10^4^ oocysts. Due to the regular size and shape of the oocysts [3], most occupy a distinct population with approximately the same FSC-A as the counting beads, but with a lower SSC-A (Fig 1A). This pattern of the SSC-A versus FSC-A scatter plot of *C*. *parvum* oocysts was previously reported [19–20]. Fluorescence intensity in the Alexa Fluor 488 channel of all events of an ungated sample is graphically represented (double lined black arrows) by a histogram (Fig 1B) and against FSC-H (Fig 1C). Using the ‘Oocyst gate’ around the major oocyst population (simple lined black arrow), the intensity of the stained oocysts can be determined (Fig 1D) and a gate set accordingly (Fig 1C); we thus could select for events no smaller than a single oocyst while including larger events that are also antibody stained (Fig 1C). Larger stained events may be clumps of oocysts or oocysts associated with debris but are not a product of non-specific binding, as confirmed using an isotype control (S1 Fig). Counting beads are easily differentiated by their high fluorescence intensity.

The appropriate gating strategy utilizes a morphology gate (SSC-A versus FSC-A, Fig 1A) and a range of fluorescence intensity (Fig 1D) followed by the selection of Alexa Fluor 488 positive events in this range of fluorescence intensity on the ungated sample (Fig 1C). Gating on a histogram, as in Fig 1B, would be unfavourable as it could include fluorescent debris. An overlay of the gated population in Fig 1C (simple lined blue arrow) on all events of an ungated sample (double lined red arrow) shows the location of Alexa Fluor 488+ events on an ungated sample (Fig 1E). Because most oocysts occupy a defined population based on morphology, the antibody provides a modest contribution to discriminating oocysts from the small number of non-oocyst events that occupy the same size and complexity as oocysts. Moreover, the antibody identifies larger events that may include oocysts.

### Quantification of an unstained sample using the PerCP channel (488 nm laser with 710/50 bandpass filter) on a BD LSRFortessa

**Fig 2:**
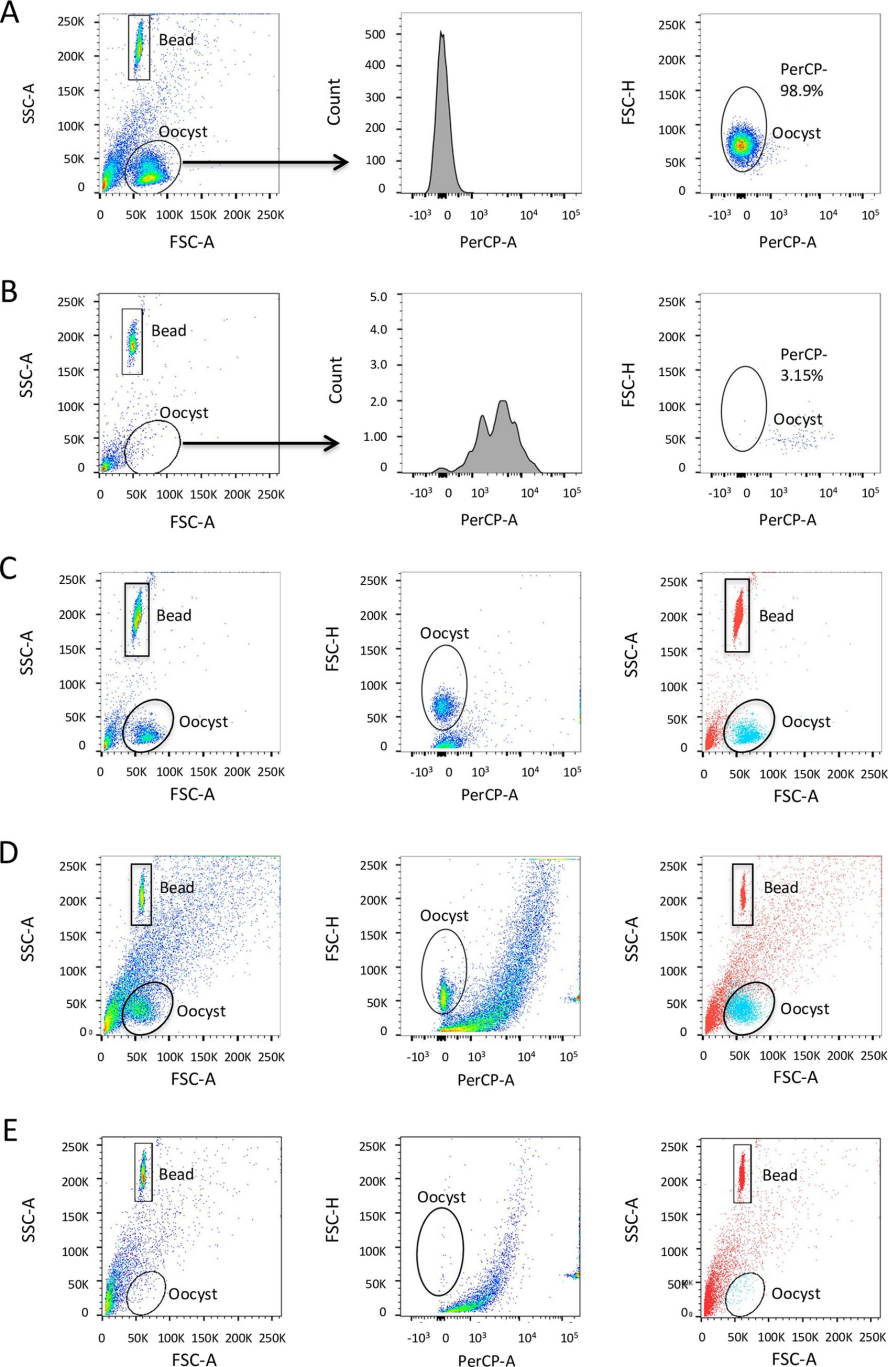
Two-step gating strategy without the use of antibody staining. (A) Pure population of *C*. *parvum* oocysts (positive control). (B) Sample from uninfected mouse intestine (negative control). (A and B) Plots from left to right: ungated sample SSC-A versus FSC-A, PerCP emission histogram of Oocyst gate, FSC-H versus PerCP including recommended PerCP gate. Representative sample from intestine (C) and from stool (D) of an infected mouse; and from stool of an uninfected mouse (E). (C, D and E) Plots from left to right: ungated sample SSC-A versus FSC-A, FSC-H versus PerCP including gate determined by positive control, overlay of PerCP- events corresponding to oocysts from the PerCP gate in blue and PerCP+ events corresponding to debris and beads in red. PerCP- events from the Oocyst gate are oocysts and PerCP+ events from the Oocyst gate are debris.

As noted, the first step of the gating strategy involves a gate surrounding the counting beads (‘Bead gate’) and another (‘Oocyst gate’) surrounding the expected population of oocysts (Fig 2A). The beads are incorporated to enable calculation of oocyst concentration in the sample. We determined that *C*. *parvum* oocysts possess a discrete fluorescence signal compared to debris. The channel that best discriminated between oocyst and non-oocyst events based on positive (Fig 2A) and negative (Fig 2B) controls is the 488 nm laser with 710/50 bandpass filter. This channel is commonly used for the fluorophore peridinin chlorophyll (PerCP). This channel afforded the narrowest peak for oocysts and better differentiated oocysts from debris in the Oocyst gate compared to other channels on the flow cytometer, although this observation was made for multiple channels in combination with the 488 nm laser. No PerCP fluorophore was used in this protocol, but for simplicity we refer to using the 488 nm laser and 710/50 bandpass filter as the PerCP channel and events gated using this channel as PerCP+ or PerCP-.

The positive control (Fig 2A) contained a population of 10^5^ purified oocysts from an intestine sample of an infected mouse; the negative control (Fig 2B) was prepared by processing the intestine of an uninfected mouse through the standard oocyst purification protocol. Oocysts in the positive control (Fig 2A) generated low emission signal when excited by the 488 nm laser and detected through a 710/50 bandpass filter compared to debris in the negative control (Fig 2B). Representative positive and negative controls were used to set the gate (‘PerCP gate’) in the PerCP channel (right panels, Fig 2A and 2B). Note that a very small percentage of events in the negative control fell within the PerCP gate. These events were captured by the Oocyst gate in the SSC-A versus FSC-A 2D-plot and are PerCP- events. We consider these events to be false positives and, when comparing counts in a pre-clinical study, we subtracted them from the number of PerCP- events in all samples. These gates are used to batch analyze all samples run in one session in the flow cytometer. A positive and negative control must be included along with samples for every analysis, as the laser intensity of the flow cytometer can vary from run to run. Noteworthy, some debris events are PerCP- and outside the Oocyst gate; using the proposed 2-step gating strategy, these events are excluded with the first gate and are not considered to be false positives as they are too small or too large to be oocysts.

Fig 2C shows results of a typical sample purified from *C*. *parvum*-infected mouse intestine. All events of the ungated sample from the left panel of Fig 2C are presented against the PerCP channel (middle panel, Fig 2C) on which the PerCP gate from the positive control (right panel, Fig 2A) is applied. The overlay of PerCP- oocysts (blue) on all events of the ungated sample shows that the PerCP+ debris and bead events (red) occupy an area distinct from the PerCP- oocysts (right panel, Fig 2C). This protocol is also suitable for *C*. *parvum* oocysts obtained from mouse feces (Fig 2D). Differences between oocyst and debris fluorescence were even more evident in samples purified from mouse feces; typically, more debris is present in fecal oocyst samples due to the abundance of vegetal material (Fig 2D). This further illustrates the importance of an appropriate negative control to estimate the potential contribution of false positives in the PerCP gate (Fig 2E).

Samples were acquired until a minimum of 5% of the counting beads had been recorded as per the manufacturer’s recommendation. We recommend collection of at least 10% of the beads for samples with high concentrations of oocysts and up to 50% of the beads for low concentrations. As the volume and concentration of counting beads in the stock solution (~ 0.2 x 10^5^ beads/20 μL, varies by lot) are known, a simple calculation provides the number of oocysts in the intestine or stool sample:

$$Number\;of\;oocysts\;per\;sample=\frac{\left(PerCP‐\;events)\;x\;(Number\;of\;beads\;in\;the\;sample\right)}{Number\;of\;beads\;acquired}$$

The result of this calculation can be multiplied by the dilution factor to obtain the oocyst concentration of the purified oocyst sample, which can be normalized by the weight of the intestine or stool sample to obtain the concentration of oocysts per gram of intestine or stool.

### Gating of unstained samples based on oocyst fluorescence in the PerCP channel is as accurate as antibody staining

**Fig 3:**
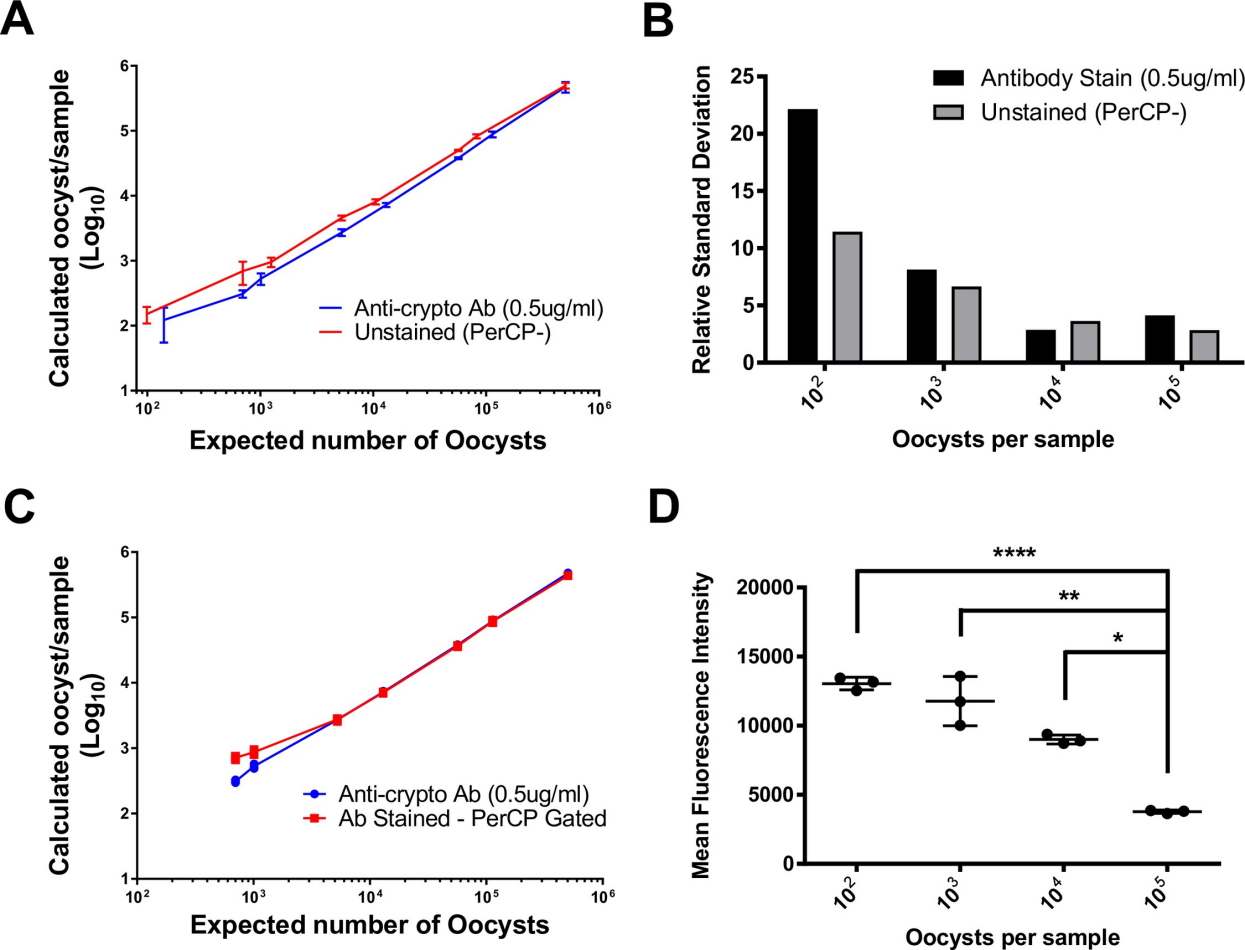
Comparison of antibody staining procedure and proposed PerCP gating strategy. (A) The counted number prepared for each sample versus the number of oocysts from each gating method. Antibody staining method (blue); unstained PerCP gating strategy (red). (B) Relative standard deviation from the antibody staining and unstained method. (C) Graph of calculated number of oocysts per sample using the antibody staining method and the PerCP gating strategy applied to stained samples. (D) Mean fluorescence intensity for oocysts stained with 0.5 μg/ml monoclonal antibody. Error bars indicate one standard deviation from the mean. * (p < 0.05); ** (p < 0.01); **** (p < 0.0001).

To confirm the accuracy of the counting method, we compared results of the PerCP gating strategy with visual counts of each sample dilution. *C*. *parvum* oocyst stock solutions were diluted and either stained with antibody or analyzed directly on the flow cytometer. After applying their respective gating strategies, the output of each method was compared with the known quantity of oocysts in each sample (Fig 3A). We determined the line of best fit for both datasets. The antibody-stained samples generated a line of best fit with the equation Y = 0.9443*X—5507 with a correlation coefficient of R^2^ = 0.9979; p<0.0001. The PerCP gating strategy generated a line of best fit with the equation Y = 0.9846*X—1102 with a correlation coefficient of R^2^ = 0.9998; p<0.0001. Quantification was most accurate in samples containing 10^3^–10^5^ oocysts and the unstained method was slightly more accurate with numbers closer to the visual counts (S1 Table). Both methods lose accuracy for samples containing < 1000 oocysts based on the relative standard deviation (Fig 3B).

To test the hypothesis that the antibody staining method was less accurate due to a loss of oocysts during the washing step of the staining procedure, we quantified oocysts in stained samples by applying the PerCP gating strategy (Fig 3C). Because the staining antibody is conjugated with Alexa 488, stained oocysts are still PerCP- and the PerCP gating strategy developed for unstained samples can be applied to stained samples to compare methods. Both analyses generated similar counts, indicating that the protocol of the staining procedure itself, and not the gating strategy, is responsible for the lower than expected counts. The two methods generated very similar results for samples containing ≥ 10^4^ oocysts.

Our second hypothesis concerns the possibility of underestimating oocyst counts at high concentrations of oocysts using the antibody staining method. A potential caveat of the antibody staining method is that at high concentrations of oocysts, the antibody may become the limiting reagent, resulting in lower mean fluorescence intensity (Fig 3D) and poorer discrimination between oocysts and debris. Using a single antibody concentration for staining resulted in a significant decrease in mean fluorescence intensity for oocyst concentrations > 10^3^ (Fig 3D). It is not possible, based on these experiments, to differentiate the two methods in terms of accuracy of oocyst quantification. However, because the PerCP gating method does not require a fluorochrome-conjugated monoclonal antibody, this strategy is less expensive; its equivalent accuracy is a factor in its favour.

### Counts from antibody staining method are lower due to loss of oocysts during antibody staining procedure

**Fig 4:**
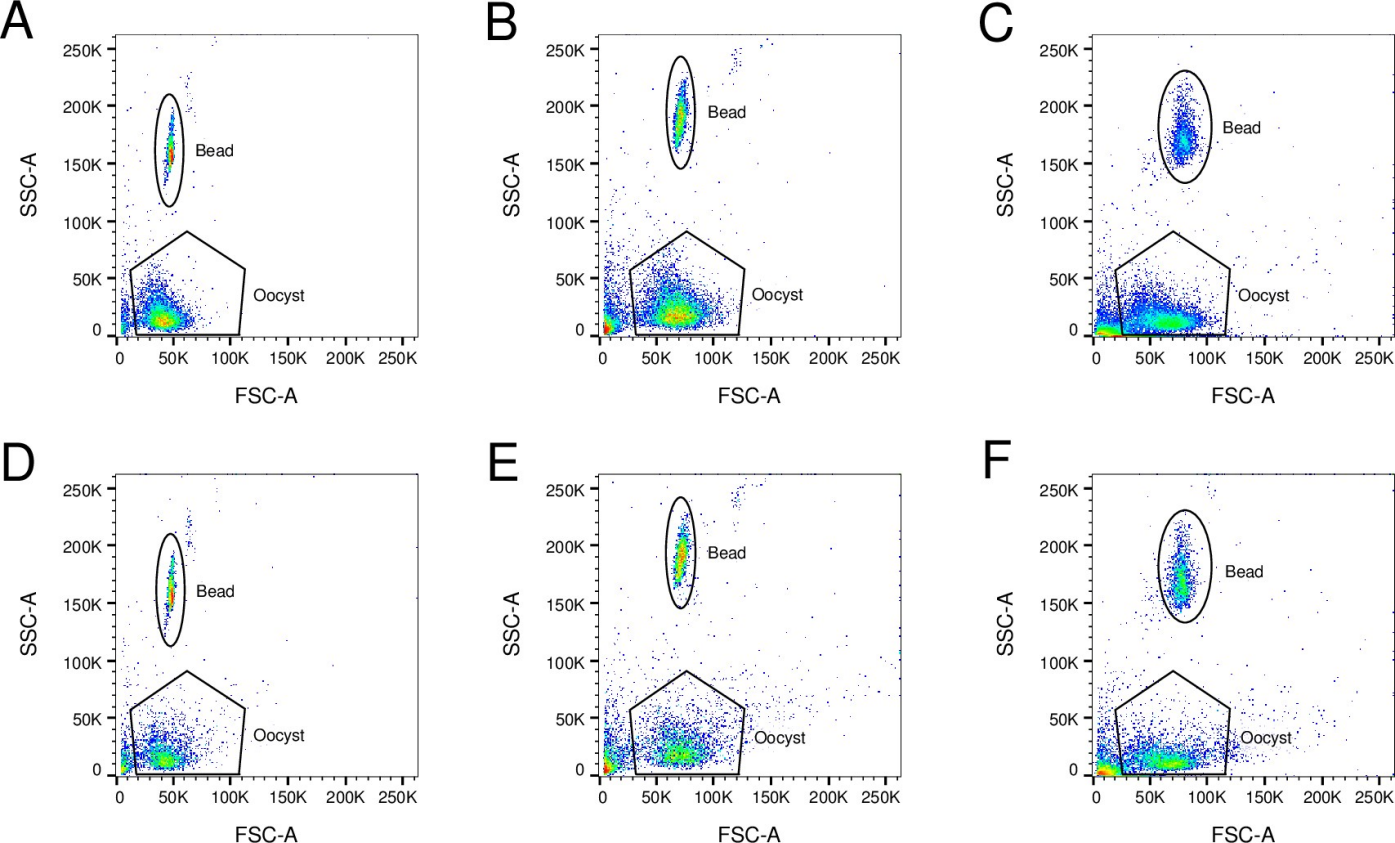
Comparison of unstained and stained samples acquired using BD LSRFortessa, BD LSRFortessa X-20 and BD FACSCanto II flow cytometers. Unstained samples from the same source acquired using BD LSRFortessa (A), BD LSRFortessa X-20 (B) and BD FACSCanto II (C) flow cytometers. Stained samples from the same source acquired using BD LSRFortessa (D), BD LSRFortessa X-20 (E) and BD FACSCanto II (F) flow cytometers. Regardless of the flow cytometer used for acquisition, approximately half of the number of events in the Oocyst gate of unstained oocysts are present in the Oocyst gate of stained oocysts due to loss during the antibody staining procedure.

Oocysts are lost during the antibody staining procedure (Fig 3C). To support our hypothesis, we conducted a second set of experiments in mice. Oocysts from each infected mouse were divided into unstained and stained samples. Because samples were from the same origin (same parasite burden) and were either stained or not, differences in oocyst counts are due to loss during staining procedure. We analyzed these samples using three flow cytometers to test the consistency of our method. Fig 4A to 4F show samples from a representative infected mouse. Fig 4A, 4B and 4C present unstained samples acquired using BD LSRFortessa, BD LSRFortessa X-20 and BD FACSCanto II flow cytometers, respectively, while Fig 4D, 4E and 4F present samples stained with Alexa 488-conjugated antibody and acquired using the same flow cytometers. Using the BD LSRFortessa, Fig 4A presents 5,659 events in the Oocyst gate, while Fig 4D shows 2,642 events in the same gate in the stained sample; 53% of the oocysts were lost during the antibody staining procedure. Analyses of data obtained using the BD LSRFortessa X-20 and BD FACSCanto instruments leads to similar conclusions: 7,173 unstained compared to 3,274 antibody stained events (representing 54% oocyst loss) and 9,057 unstained compared to 4,397 antibody stained (51% oocyst loss), respectively. The raw numbers represent events shown in Fig 4A to 4F and vary slightly between flow cytometers only because they are different samples from the same source. The number of oocysts lost during the staining procedure varied between samples, but 50% oocyst loss was typical (S2 Fig).

### Efficacy of antibody staining is inversely proportional to oocyst concentration in samples

**Fig 5:**
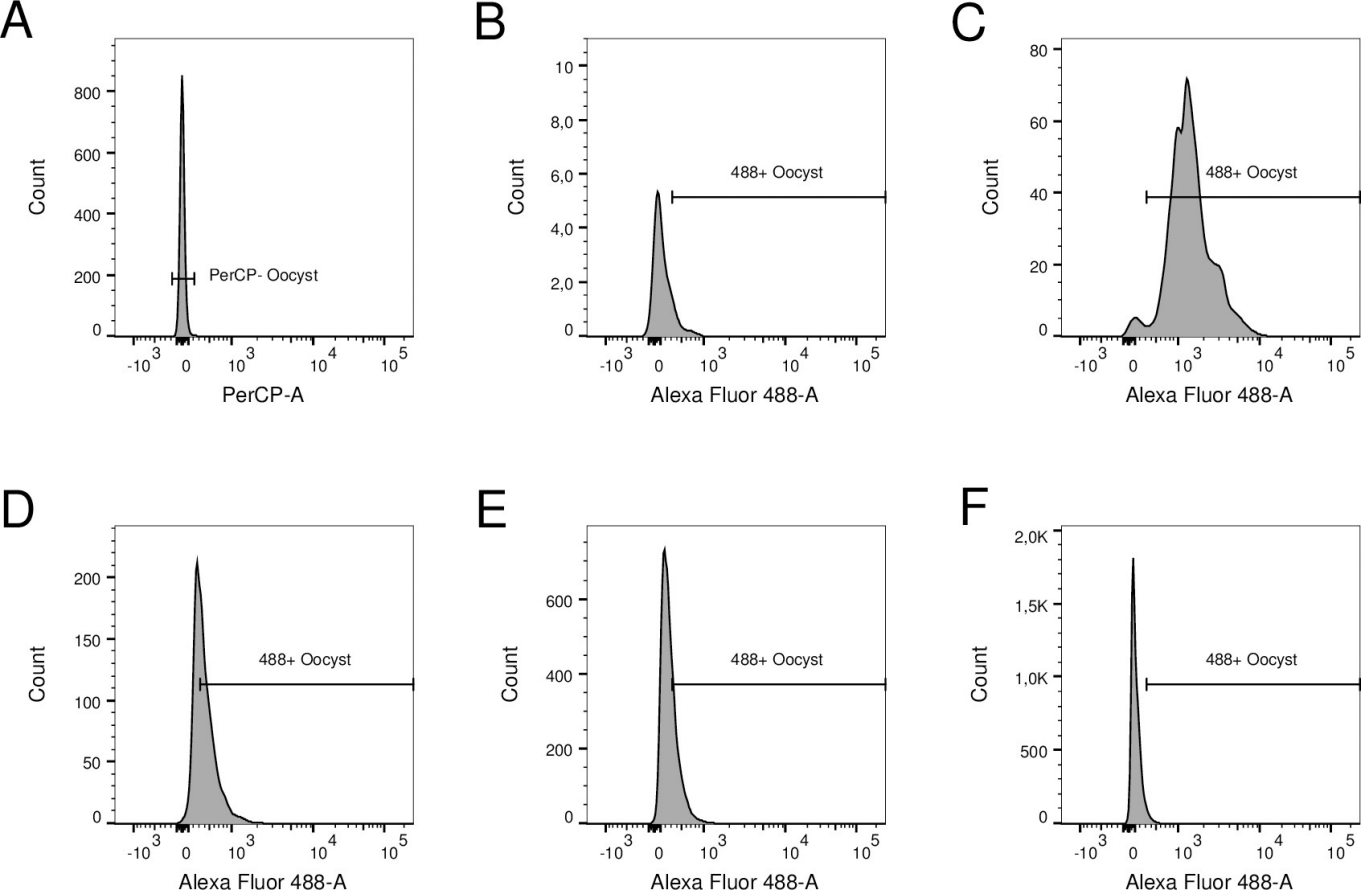
Comparison of staining efficacy of samples acquired using BD LSRFortessa flow cytometer. (A) PerCP fluorescence of unstained oocysts from an infected mouse. Alexa 488 fluorescence of stained sample from uninfected mouse (B) or stained samples from infected mice (C to F). Increasing levels of parasite burdens (counts in Y axis) show decreasing antibody staining efficacy: > 90% (C); < 90% but > 50% (D); < 50% but > 25% (E); < 25% (F).

Fig 5A shows a representative histogram of the PerCP channel of unstained oocysts from the Oocyst gate (second step of the proposed 2-step gating strategy for unstained samples). Fig 5B to 5F show fluorescence in the Alexa 488 channel of stained events from the Oocyst gate for increasing numbers of oocysts stained with a standard antibody concentration of 0.5 ug/ml (second step of the 2-step gating strategy for stained samples). An antibody stained sample containing no oocysts (Fig 5B) shows the events corresponding to false positives (total number of Alexa 488+ events = 10). Samples in Fig 5C to 5F came from infected mice. In Fig 5C, 2,555 oocysts were counted in the Oocyst gate of which 97% (2,479) were Alexa 488+. Increasing the oocyst concentration in the sample, only 63% (2,235) of the 3,555 oocysts in the Oocyst gate were Alexa 488+ in Fig 5D. Continuing this trend, of 10,310 oocysts in the Oocyst gate, only 3,362 (33%) were Alexa 488+ in Fig 5E and of 12,544 oocysts in the Oocyst gate, only 571 (5%) were Alexa 488+ in Fig 5F. The staining efficacy was independent of the flow cytometer used for acquisition and data was similar for the BD LSRFortessa X-20 and BD FACSCanto II flow cytometers (S3 Fig and S4 Fig).

**Fig 6:**
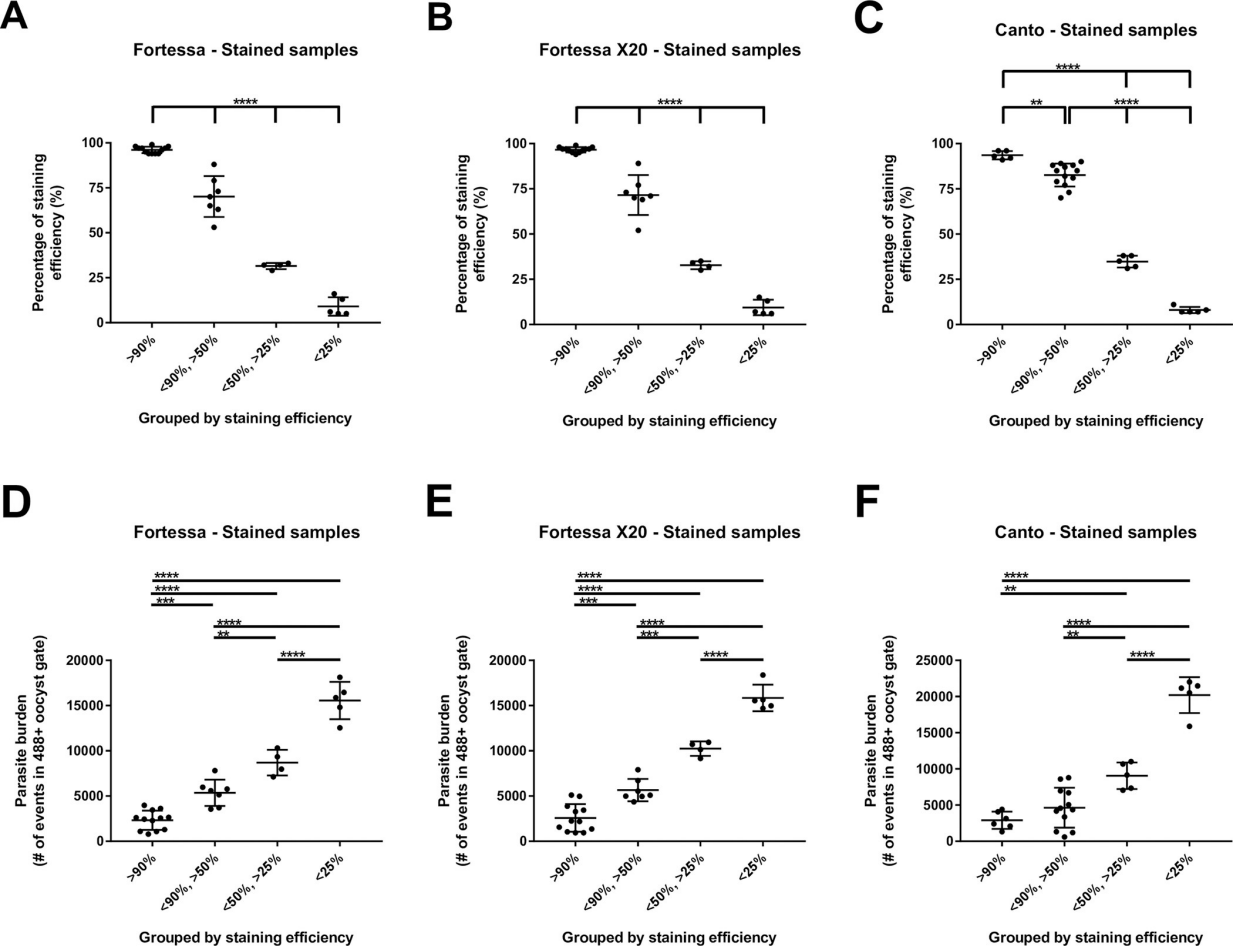
Comparison of relationship between staining efficacy and parasite burden in samples acquired using BD LSRFortessa, BD LSRFortessa X-20 or BD FACSCanto II flow cytometer. Samples acquired with BD LSRFortessa (A), BD LSRFortessa X-20 (B) or BD FACSCanto II (C) flow cytometer were divided by percentage of Alexa 488+ events among events in Oocyst gate (staining efficacy). Parasite burden of samples categorized previously by staining efficacy and acquired with BD LSRFortessa (D), BD LSRFortessa X-20 (E) or BD FACSCanto II (F) flow cytometer. Staining efficacy (A to C) is inversely proportional to parasite burden (D to F) in same samples. ** (p < 0.01); *** (p < 0.001); **** (p < 0.0001).

To better define the relationship between antibody staining and parasite burden, we divided samples from intestines of infected mice according to staining efficacy, defined as % Alexa 488+ samples among events in the Oocyst gate. All events in the Oocyst gate were considered oocysts because, for all corresponding unstained samples, 99% (95%-100%) of events in the Oocyst gate were PerCP- (second step of the proposed 2-step gating strategy for unstained samples). Therefore, samples showing < 90% Alexa 488 staining efficacy underestimated the oocyst count (second step of the 2-step gating strategy for stained samples). Stained samples were divided into four categories: > 90% Alexa 488 staining efficacy; < 90%—> 50%; < 50%—> 25%; or < 25%. Fig 5C, 5D, 5E and 5F are representative samples in these four categories, respectively. Data for samples from intestines of all infected mice were divided in these four categories (Fig 6A, 6B and 6C). No difference in staining efficacy was seen among samples analyzed with the BD LSRFortessa (Fig 6A), BD LSRFortessa X-20 (Fig 6B) or BD FACSCanto II (Fig 6C) flow cytometers. Parasite burdens in these samples are graphically presented (Fig 6D, 6E and 6F). Again, no differences were seen in parasite burden from samples analyzed with the BD LSRFortessa (Fig 6D), BD LSRFortessa X-20 (Fig 6E) or BD FACSCanto II (Fig 6F) flow cytometers. There is an obvious relationship between staining efficacy (Fig 6A, 6B and 6C) and parasite burden (Fig 6D, 6E and 6F); Alexa 488 staining efficacy was inversely proportional to intestinal parasite burden in samples from infected mice.

## Discussion

Flow cytometry hinges on the ability to discriminate a population of particles of interest from debris, confounding events and artefacts. Normally, this is done using fluorophore-conjugated antibodies specific for epitopes on desired targets and populations. Much work has been published on methods for the identification of *Cryptosporidium spp*. using anti-*C*. *parvum* oocyst antibodies [17]. These methods (2-step gating strategy for stained samples, Fig 1A to 1E) have been compared exhaustively [21–22] and share a major characteristic: they are designed for reliable detection of *Cryptosporidium spp*., but not for quantification of parasite burden. Through our initial flow cytometry work with *C*. *parvum*, we observed innate differences between emission spectra (proposed 2-step gating strategy for unstained samples, Fig 2A and 2B) between oocysts and intestinal and fecal debris (Fig 2C and 2D). These innate characteristics enabled discrimination of oocysts from debris with the same accuracy as antibody staining (Fig 3A and 3B).

By removing the need to stain the sample, we eliminated the cost and troubleshooting associated with the antibody. More importantly, this increased accuracy of oocyst counts. For research projects in which parasite burdens are compared among different groups, the washing step required during the antibody staining protocol is likely to lower oocyst counts and reduce the reliability of the counts (Fig 3C and Fig 4A to 4F). The method we describe here is well-suited for research purposes, particularly for animal studies focused on measuring the outcome of treatment or prevention of *Cryptosporidium spp*. infection. In the experimental mouse model used in our laboratory, we experience > 5 orders of magnitude difference in oocyst burden between experimental groups [15]. The use of a standard concentration of antibody would either over-stain low burden samples, a waste of money and resources, or under-stain high burden samples (Fig 3D). We demonstrated that antibody staining efficacy is inversely proportional to parasite burden; at low oocyst concentrations, the antibody method reaches > 90% efficacy while at high oocyst concentrations, the antibody becomes the limiting factor and staining efficacy progressively decreases to 5% (Fig 5A to 5F and Fig 6A to 6F). That staining efficacy was inversely proportional to intestinal parasite burden in samples of infected mice (Fig 6A to 6F) is the main disadvantage of antibody staining. It is then necessary to estimate parasite burden by optical microscopy before staining oocysts to provide an adequate amount of antibody during the staining protocol; this step reduces the usefulness of antibody staining for oocyst counting by flow cytometry. The proposed method to measure oocyst burden without staining allows direct analysis of samples by flow cytometry without the need to consider the number of oocysts in each sample beforehand.

Small details (homogenization of intestine and optical density of the saturated solution used during the purification process) can influence the abundance of debris in purified oocyst samples. Also, the protocol used to purify oocysts may not exclude or eliminate bacteria. Optimizing the sample purification protocol is therefore an essential step to decrease confounding factors and false positives. We recommend following the purification protocol described here and to confirm the purity of a sub-set of samples by microscopy before counting oocysts by flow cytometry. It is advisable to subtract the average count of the negative (uninfected) controls from the counts of other samples as a baseline, with the expectation that the proportion of false positives is consistent among samples regardless of oocyst concentration. A positive control of a pure population of oocysts should also always be included to set the initial morphology gate. For intestine and stool samples from experimentally-infected mice, this technique offers a simple and less expensive method to quantify parasite burden. We do not recommend this technique for clinical samples as an extra level of identification through antibody staining is necessary.

### Conclusion

The proposed 2-step gating strategy for unstained samples exploits the typical morphology of oocysts in an SSC-A versus FSC-A plot and the absence of fluorescence of oocysts in multiple channels using the 488 nm laser. This protocol is fast, reliable, does not require optimization of antibody concentrations and is as accurate as published methods using anti-*C*. *parvum* antibody staining, with the bonus of requiring fewer resources. Our method is designed mainly for rapid, high-throughput quantification of oocysts and has the potential to promote drug and vaccine discovery for control of cryptosporidiosis. Research on *Cryptosporidium* spp. is becoming a priority; new methods such as this protocol could accelerate research in many laboratories.

## Supporting information

S1 Table Mean and standard deviation of the calculated number of oocysts from each gating method for each number of oocysts. Equation for line of best fit with Pearson correlation coefficient.
(TIF)

S1 Fig Alexa Fluor 488+ events detected outside the oocyst gate as a percentage of total oocysts in the sample. This analysis excludes debris smaller than the oocyst gate by morphology (SSC-A vs FSC-A).
(TIF)

S2 Fig Comparison of oocyst lost during washes of stained samples acquired using BD LSRFortessa, BD LSRFortessa X-20 and BD FACSCanto II flow cytometers. (TIF)

S3 Fig Comparison of staining efficacy of samples acquired using BD LSRFortessa X-20 flow cytometer. (A) PerCP fluorescence of unstained oocysts from an infected mouse. Alexa 488 fluorescence of stained sample from uninfected mouse (B) or stained samples from infected mice (C to F). Increasing levels of parasite burdens (counts in Y axis) show decreasing antibody staining efficacy: > 90% (C); > 50% but < 90% (D); > 25% but < 50% (E); < 25% (F).
(TIF)

S4 Fig Comparison of staining efficacy of samples acquired using BD FACSCanto II flow cytometer. (A) PerCP fluorescence of unstained oocysts from an infected mouse. Alexa 488 fluorescence of stained sample from uninfected mouse (B) or stained samples from infected mice (C to F). Increasing levels of parasite burdens (counts in Y axis) show decreasing antibody staining efficacy: > 90% (C); > 50%—< 90% (D); > 25%—< 50% (E); < 25% (F).
(TIF)
