# Supplementary figures and images for: A protocol to count Cryptosporidium oocysts by flow cytometry without antibody staining
Source: PLoS Negl Trop Dis. 2019 Mar 20;13(3):e0007259. doi: 10.1371/journal.pntd.0007259 (PMC6443187; doi:10.1371/journal.pntd.0007259)

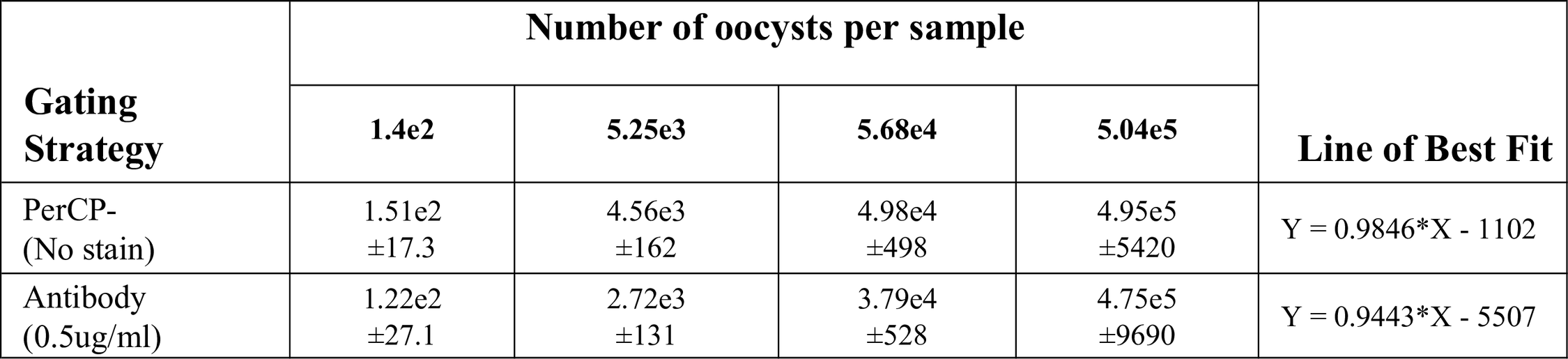

Supplement: S1 Table — Equation for line of best fit with Pearson correlation coefficient. (TIF) [file pntd.0007259.s001.tif]

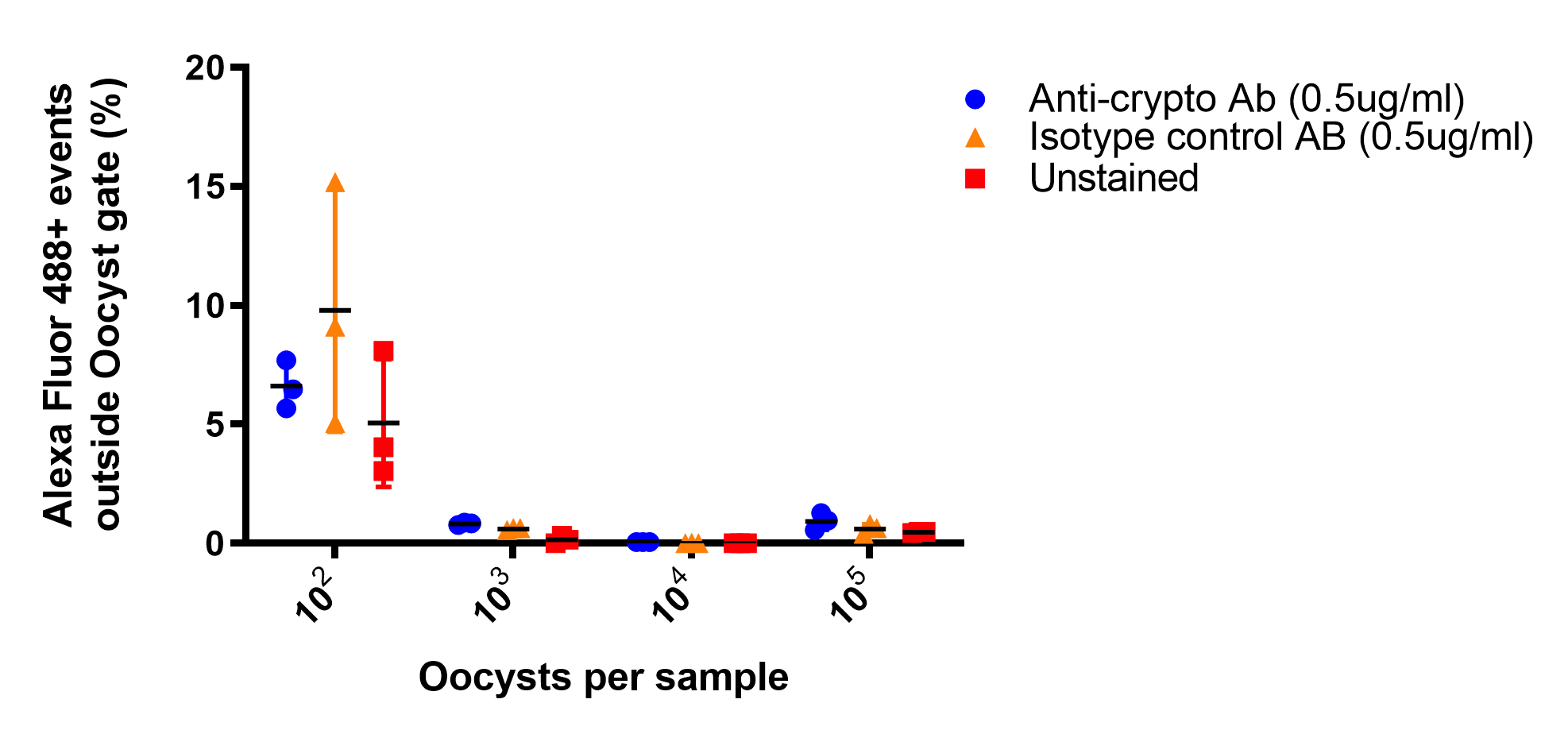

Supplement: S1 Fig — This analysis excludes debris smaller than the oocyst gate by morphology (SSC-A vs FSC-A). (TIF) [file pntd.0007259.s002.tif]

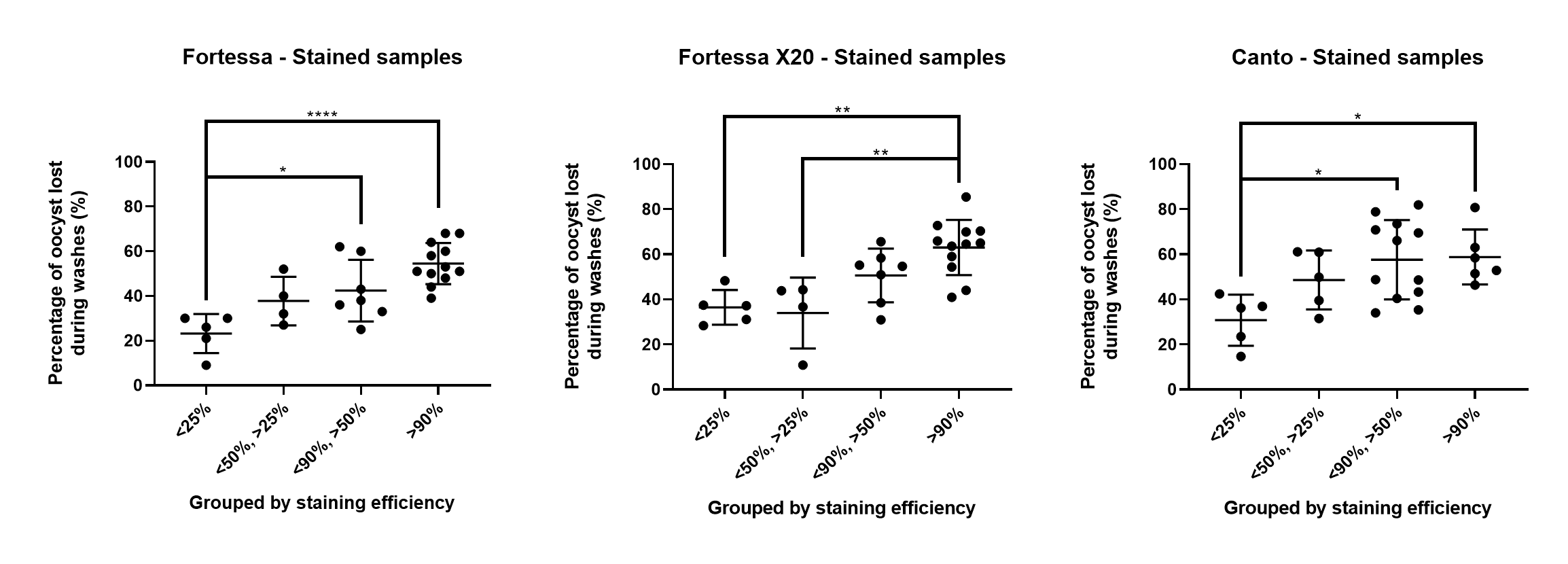

Supplement: S2 Fig — (TIF) [file pntd.0007259.s003.tif]

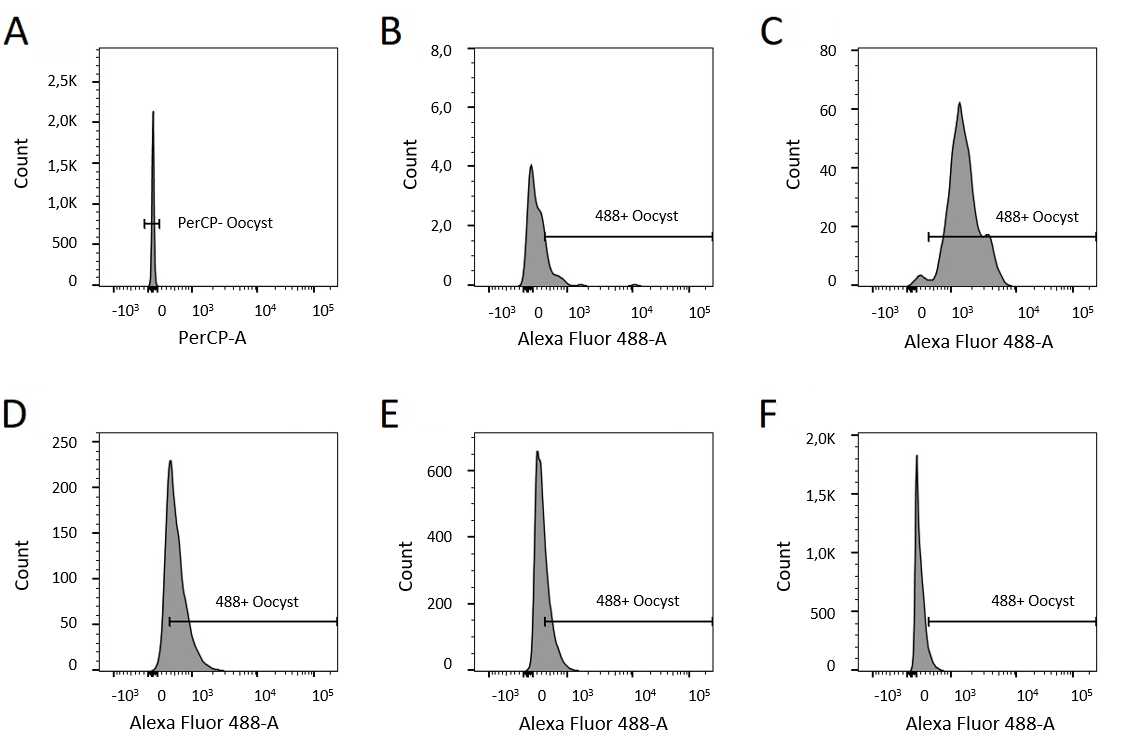

Supplement: S3 Fig — (A) PerCP fluorescence of unstained oocysts from an infected mouse. Alexa 488 fluorescence of stained sample from uninfected mouse (B) or stained samples from infected mice (C to F). Increasing levels of parasite burdens (counts in Y axis) show decreasing antibody staining efficacy: > 90% (C); > 50% but < 90% (D); > 25% but < 50% (E); < 25% (F). (TIF) [file pntd.0007259.s004.tif]

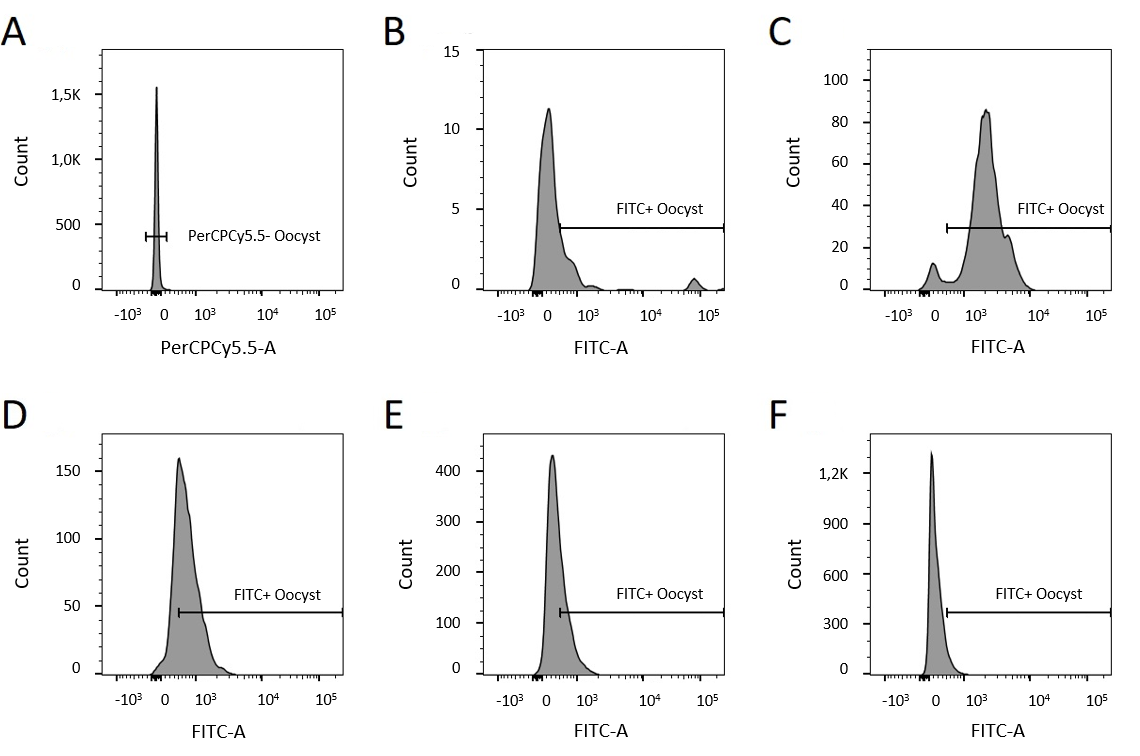

Supplement: S4 Fig — (A) PerCP fluorescence of unstained oocysts from an infected mouse. Alexa 488 fluorescence of stained sample from uninfected mouse (B) or stained samples from infected mice (C to F). Increasing levels of parasite burdens (counts in Y axis) show decreasing antibody staining efficacy: > 90% (C); > 50%—< 90% (D); > 25%—< 50% (E); < 25% (F). (TIF) [file pntd.0007259.s005.tif]
